# CYP2D6 in the Brain: Potential Impact on Adverse Drug Reactions in the Central Nervous System—Results From the ADRED Study

**DOI:** 10.3389/fphar.2021.624104

**Published:** 2021-05-07

**Authors:** Katja S. Just, Harald Dormann, Mathias Freitag, Marlen Schurig, Miriam Böhme, Michael Steffens, Catharina Scholl, Thomas Seufferlein, Ingo Graeff, Matthias Schwab, Julia C. Stingl

**Affiliations:** ^1^ Institute of Clinical Pharmacology, University Hospital of RWTH Aachen, Aachen, Germany; ^2^ Central Emergency Department, Hospital Fürth, Fürth, Germany; ^3^ Department of Geriatric Medicine, University Hospital of RWTH Aachen, Aachen, Germany; ^4^ Research Department, Federal Institute of Drugs and Medical Devices, Bonn, Germany; ^5^ Internal Medicine Emergency Department, Ulm University Medical Centre, Ulm, Germany; ^6^ Interdisciplinary Emergency Department (INZ), University Hospital of Bonn, Bonn, Germany; ^7^ Dr. Margarete Fischer-Bosch-Institute of Clinical Pharmacology, Stuttgart, Germany; ^8^ Department of Clinical Pharmacology, University of Tuebingen, Tuebingen, Germany; ^9^ Department of Pharmacy and Biochemistry, University of Tuebingen, Tuebingen, Germany

**Keywords:** CYP2D6, pharmacogenetics, dizziness, central nervous system, brain, older adults, adverse drug reaction

## Abstract

Cytochrome P450 (CYP) 2D6 is a polymorphic enzyme expressed in the central nervous system (CNS), important in drug metabolism and with a potentially constitutive role in CNS function such as vigilance. This study aimed to analyze variability in CYP2D6 activity linked to vigilance-related adverse drug reactions (ADRs) in the CNS. A dataset of N = 2939 ADR cases of the prospective multicenter observational trial in emergency departments (EDs) (ADRED; trial registration: DRKS-ID: DRKS00008979) was analyzed. Dizziness as the most frequent reported CNS ADR symptom (12.7% of patients, *n* = 372) related to vigilance was chosen as the outcome. The association of dizziness with CYP2D6 activity markers was analyzed. The number of CYP2D6 substrates taken, a CYP2D6 saturation score (no, moderate, and strong saturation), a CYP2D6 saturation/inhibition score (no, weak, moderate, and strong), and composed CYP2D6 activity using a genotyped subsample (*n* = 740) calculating additive effects of genotype and CYP2D6 saturation by drug exposure were used as CYP2D6 activity markers. Effects were compared to other frequent nonvigilance-related CNS ADR symptoms (syncope and headache). Secondary analyses were conducted to control for other ADR symptoms frequently associated with dizziness (syncope, nausea, and falls). The majority of all patients (64.5%, *n* = 1895) took at least one drug metabolized by CYP2D6. Around a third took a CNS drug (32.5%, *n* = 955). The chance to present with drug-related dizziness to the ED increased with each CYP2D6 substrate taken by OR 1.11 [1.01–1.23]. Presenting with drug-related dizziness was more likely with CYP2D6 saturation and saturation/inhibition (both OR 1.27 [1.00–1.60]). The composed CYP2D6 activity was positively associated with dizziness (*p* = 0.028), while poorer activity affected patients more often with dizziness as an ADR. In contrast, nonvigilance-related ADR symptoms such as syncope and nausea were not consistently significantly associated with CYP2D6 activity markers. This study shows an association between the number of CYP2D6 substrates, the predicted CYP2D6 activity, and the occurrence of dizziness as a CNS ADR symptom. As dizziness is a vigilance-related CNS symptom, patients with low CYP2D6 activity might be more vulnerable to drug-related dizziness. This study underlines the need for understanding individual drug metabolism activity and individual risks for ADRs.

## Introduction

Central nervous system (CNS) drugs are often substrates of the phase-I enzyme cytochrome P450 (CYP) 2D6. Due to pharmacogenetic polymorphisms in CYP2D6, drug clearances may individually vary, thereby affecting drug efficacy and safety (Stingl and Viviani, 2015). Translation of pharmacogenetic knowledge into clinic is promised to increase drug safety by individualizing drug treatment and preventing adverse drug reactions (ADRs) (Evans and Relling, 2004). Patients metabolizing CYP2D6 substrates slower (e.g., poor metabolizers) or in the case of a prodrug faster (ultrarapid metabolizers) than others are usually expected to be more vulnerable to dose-related ADRs when taking CYP2D6 substrates such as amitriptyline, metoprolol, or codeine (Ingelman-Sundberg et al., 2007). However, pharmacogenetic variability does not fully explain individual differences in drug exposure (Matthaei et al., 2015). CYP2D6 is not inducible but can be inhibited by several factors, including drug–drug interactions and disease-related factors (Ingelman-Sundberg et al., 2007). This might lead to a different CYP2D6 phenotype compared to the genotype-predicted phenotype. This phenomenon is called phenoconversion or phenocopying (Klomp et al., 2020).

Since CYP2D6 is also expressed in the brain, pharmacogenetic variability might influence the CNS’s vulnerability to ADRs (Stingl et al., 2013). CYP enzymes could play a constitutive role in the brain as they are involved in metabolism of endogenous substrates and thereby contribute to the biochemical homeostasis of the brain (Stingl et al., 2013). Polymorphisms in CYP2D6 are associated with variability in vigilance, sustained attention, and alertness as shown in functional magnetic resonance imaging (fMRI) studies during working and emotional tasks and in resting states (Kirchheiner et al., 2011; Stingl et al., 2012; Viviani et al., 2020).

ADRs and their consequences have a substantial impact on the health-care system. Around 6.5% of all unplanned emergency department (ED) admissions are likely to be caused by ADRs (Lazarou et al., 1998; Pirmohamed et al., 2004; Schurig et al., 2018), often affecting older and multi-medicated adults (Hanlon et al., 1997; Just et al., 2020a). These patients seem to be especially vulnerable to drug-related symptoms of the CNS (Just et al., 2020c), and drugs acting within the CNS often get suspected for causing an ADR (Just et al., 2020a). Frequent symptoms related to CNS drugs in older patients include syncope and falls, most likely due to their sedative properties (Stingl et al., 2020).

The aim of this study was to analyze the role of CYP2D6 activity predicted by drug exposure and, if available, by genotype on CNS ADRs presenting to the ED connected to vigilance.

## Materials and Methods

### Study Design and Population

Data of the multicenter observational study “Adverse Drug Reactions in Emergency Departments” (ADRED; trial registration: DRKS-ID: DRKS00008979) were analyzed. The ADRED study collects cases of ADRs that led to unplanned presentations to four large EDs of tertiary care and academic teaching hospitals in Germany. Within those hospitals, the prevalence of ADRs was shown to be 6.5% among all ED admissions (Schurig et al., 2018). Further information on study design and enrollment is published elsewhere (Schurig et al., 2018; Just et al., 2020a).

Adults presenting with symptoms, which were seen in a possible, probable, or certain relation to a drug after standardized causality assessment by study personnel using the WHO–Uppsala Monitoring Centre system were enrolled (definition of an ADR) (Uppsala Monitoring Centre, 2013). The study personnel consisted of trained physicians and pharmacists. Demographical and clinical data were collected. All patients provided written informed consent. Cases enrolled between December 2015 and December 2018 (first-funding phase) were included in the analysis. The study was approved by the responsible ethical committee of the University of Bonn (202/15).

### Classification of Drugs

The current drug intake was documented. Every drug taken was separately assessed with a standardized causality assessment by the study personnel for causal relation to the ADR (Uppsala Monitoring Centre, 2013). Thus, drugs were discriminated in suspicion for causing an ADR or not. For this analysis, drugs were additionally classified into intended CNS efficacy (CNS drug: yes, no), being a major substrate of CYP2D6 (CYP2D6 described as the main metabolic pathway: yes, no), and being a minor substrate of CYP2D6 (CYP2D6 as one of several enzymes involved in metabolism: yes, no). Major and minor CYP2D6 substrates were then combined in one classification group (CYP2D6 substrate). We classified all drugs assessed as possibly having caused an ADR in at least 0.3% of all cases and those that were taken by at least 3% of all patients irrespective of being causative or not. The hereby identified drugs were independently classified by a clinical pharmacologist and a clinical pharmacist using information of drug labels and databases such as UpToDate (www.uptodate.com), DrugBank (www.drugbank.com), or PharmGKB (www.pharmgkb.org). Discrepancies were discussed and a consensus was established. Respective lists can be found in the supplement (Supplementary Table S1).

Assuming CYP2D6 enzyme activity saturation by the intake of more than two CYP2D6 substrates (Sindrup et al., 1992; Taylor et al., 2006), we calculated a simple CYP2D6 saturation score. Therefore, we defined no CYP2D6 saturation as not taking any CYP2D6 substrate at all, moderate CYP2D6 saturation as taking one or two CYP2D6 substrates, and strong saturation as taking three or more CYP2D6 substrates.

Further, we calculated a score combining CYP2D6 saturation with enzyme inhibition. Therefore, substrates were calculated with one point as described above. In addition, those drugs known to be clinical inhibitors of CYP2D6 used in drug interaction studies and drug labeling (FDA, 2020) were calculated in the following way: a weak inhibitor was weighted with one point, a moderate inhibitor with two, and a strong inhibitor with three points. Thus, we calculated a CYP2D6 saturation/inhibition score adding up the individual substrate and inhibitor exposure.

### Classification of Symptoms

All symptoms seen on ED arrival in the context of ADR were documented. Symptoms were classified using the hierarchy of the Medical Dictionary for Regulatory Activities (MedDRA) (Wood, 1994). In this hierarchy, low-level terms (LLTs) are connected to preferred terms (PTs) that are again connected to the affected system organ classes (SOCs). We analyzed symptoms on the PT level and used the SOC level for identifying common symptoms affecting the CNS (SOCs: nervous system and psychiatric disorders).

The most frequent CNS symptoms were dizziness (12.7%, *n* = 372), syncope (6.2%, *n* = 183), headache (3.9%, *n* = 116), confusion (1.4%, *n* = 40), seizure (1.3%, *n* = 38), and paresthesia (1.3% *n* = 38). We determined dizziness as the most common CNS symptom with association to vigilance. Analyses on the second and third most frequent CNS symptoms syncope and headache were included as a control testing for nonvigilance-related CNS ADR symptoms. As patients presented in median with two symptoms (Just et al., 2020c), we conducted sensitivity analyses with ADR symptoms frequently associated with dizziness.

### Genotyped Subsample

A biosample, either blood or mucosa, was taken from a subsample of the total population for pharmacogenetic analyses after informed consent. This subsample consisted of patients cognitively fit to give informed consent that were available for the study personnel during their ED visit or the following hospitalization. Genomic DNA was isolated from the biosamples using magnetic beads with the MagNA PureLC DNA Isolation Kit-Large Volume (Roche Diagnostics GmbH., Mannheim, Germany). The iPLEX®PGx 74 Panel together with the VeriDose™ CYP2D6 CNV Panel (both Agena Bioscience, Inc., San Diego, CA, United States) were used for pharmacogenetic analyses. The VeriDose™ CYP2D6 CNV Panel detected copy number variations (CNVs) for CYP2D6, even in the presence of nonfunctional hybrid alleles, including *36, *13, and *68. The iPLEX®PGx 74 Panel analyses 69 single nucleotide polymorphisms in 20 pharmacogenes. In addition to CYP2D6, we characterized other polymorphic CYP enzymes CYP2C9 and CYP2C19. The characterization of this cohort and the phenotyping methods are published elsewhere (Just et al., 2020b). We used the common phenotypes classification such as ultrarapid metabolizers (UM), rapid metabolizers (RMs), normal metabolizers (NMs), intermediate metabolizers (IMs), and poor metabolizers (PMs).

Within this subsample genotyped for CYP2D6 activity, we analyzed the additive effects of the CYP2D6 genotype and drug exposure of CYP2D6 substrates. For calculation of additive effects of CYP2D6 saturation by drugs, we modified the phenotype score in the following way: a PM kept poor activity. All other phenotypes were classified into the next lower activity phenotype in the case of moderate CYP2D6 saturation by drug intake as described above. For example, a person classified originally as CYP2D6 NM based on the genotype got classified as intermediate activity when taking one or two CYP2D6 substrates and classified as poor activity when taking three or more CYP2D6 substrates concomitantly (Supplementary Table S2).

### Statistical Analyses

Continuous parameters were presented as median with interquartile range (IQR) and categorical parameters in absolute numbers and respective percentages. Patient characteristics of those presenting either with dizziness or without were compared using chi-squared test for categorical and Mann–Whitney test for continuous variables. Associations of dizziness with CYP2D6 activity markers, number of CYP2D6 substrate intake, the CYP2D6 saturation score, the CYP2D6 saturation/inhibition score, and composed CYP2D6 activity were analyzed using unadjusted binary logistic regression analyses for CYP2D6 substrate intake and the Mantel–Haenszel test of trend for CYP2D6 saturation and CYP2D6 saturation/inhibition scores, as well as for composed CYP2D6 activity in the genotyped subgroup.

Assuming a different effect in terms of a negative control in other frequent CNS symptoms of CYP2D6 activity on nonvigilance-related ADR symptoms, we repeated these analyses with syncope and headache. To control for effects of correlating dizziness, we excluded patients with dizziness in a sensitivity analysis.

In addition, we tested associations of CYP2D6 activity markers (number of CYP2D6 substrates taken, CYP2D6 saturation and CYP2D6 saturation/inhibition scores, and composed CYP2D6 activity) with symptoms frequently associated with dizziness to control for other symptoms that might influence the effect of dizziness. Hereby, we excluded patients with dizziness likewise.

In the last step, we adjusted the effect of CYP2D6 activity markers (number of CYP2D6 substrates taken, CYP2D6 saturation and CYP2D6 saturation/inhibition scores, and composed CYP2D6 activity) on the occurrence of dizziness using binary logistic regression analyses. To this end, we tested the absolute number of CYP2D6 substrates taken continuously. For the analyses of the CYP2D6 saturation and CYP2D6 saturation/inhibition scores, we summarized the scores (any CYP2D6) of saturation or saturation/inhibition (weak, moderate, and strong) vs. no CYP2D6 saturation or saturation/inhibition by drug exposure. For analyses of composed CYP2D6 activity, we summarized low activity (intermediate and poor) vs. higher activity (normal and ultrarapid). We tested two models. The first model was adjusted for age and sex, and the second model for age, sex, and the total number of taken drugs irrespective of being a CYP2D6 substrate and thereby adjusting for polypharmacy. Odds ratios (ORs) with corresponding 95% confidence intervals (CI) are shown.

A *p*-value < 0.05 was considered statistically significant. A *p*-value < 0.1 gets reported as a tendency toward significance. All statistical analyses were conducted using IBM® SPSS® Statistics (Version 25, IBM Inc., Armonk, NY, United States).

## Results

In total, N = 2939 patients were enrolled that presented to the ED due to an ADR. The majority of all patients (64.5%, *n* = 1895) took at least one drug metabolized by CYP2D6, with 54.5% (*n* = 1337) taking at least one major CYP2D6 substrate, 40.8% (*n* = 1199) taking at least one minor CYP2D6 substrate, and 10.8% (*n* = 316) taking at least one CYP2D6 inhibitor. Around a third of all patients took at least one drug with intended CNS efficacy (CNS drug, 32.5%, *n* = 955). Intake frequencies of single CYP2D6 substrates and drugs with intended CNS efficacy are shown in Table 1 **.**

**TABLE 1:** Intake frequencies of CYP2D6 substrates and drugs with intended CNS efficacy per CNS symptoms with unadjusted *p*-values.

**Substance**	**Total population (N = 2939), n (%)**	**Patients with dizziness (*n* = 372), n (%)**	***p*-value comparing patients with dizziness and without**	**Patients with syncope (*n* = 183), n (%)**	***p*-value comparing patients with syncope and without**	**Patients with headache (*n* = 116), n (%)**	***p*-value comparing patients with headache and without**
**CYP2D6 substrates only**							
Amiodarone	57 (1.9)	12 (3.2)	0.054	6 (3.3)	0.175	1 (0.9)	0.391
Bisoprolol	286 (9.7)	47 (12.6)	**0.043**	25 (13.7)	0.064	10 (8.6)	0.681
Carvedilol	96 (3.3)	21 (5.6)	**0.006**	10 (5.5)	0.084	1 (0.9)	0.137
Metoprolol	932 (31.7)	122 (32.8)	0.631	62 (33.9)	0.515	12 (10.3)	**<0.001**
Simvastatin	610 (20.8)	95 (25.5)	**0.015**	51 (27.9)	**0.014**	12 (10.3)	**0.005**
Tamsulosin	221 (7.5)	29 (7.8)	0.829	14 (7.7)	0.945	4 (3.4)	0.090
Tiotropiumbromide	147 (5.0)	17 (4.6)	0.683	6 (3.3)	0.269	1 (0.9)	**0.037**
**CYP2D6 substrates with CNS efficacy**							
Amitriptyline	69 (2.3)	9 (2.4)	0.922	3 (1.6)	0.513	5 (4.3)	0.154
Duloxetine	33 (1.1)	5 (1.3)	0.665	1 (0.5)	0.445	2 (1.7)	0.531
(Es) Citalopram	143 (4.9)	14 (3.8)	0.290	10 (5.5)	0.697	6 (5.2)	0.875
Metoclopramide	102 (3.5)	12 (3.2)	0.783	3 (1.6)	0.162	3 (2.6)	0.595
Mirtazapine	139 (4.7)	18 (4.8)	0.915	9 (4.9)	0.901	4 (3.4)	0.507
Oxycodone	100 (3.4)	13 (3.5)	0.916	2 (1.1)	0.075	0 (0)	**0.039**
Quetiapine	59 (2.0)	4 (1.1)	0.170	3 (1.6)	0.714	1 (0.9)	0.369
Risperidone	57 (1.9)	4 (1.1)	0.196	9 (4.9)	**0.003**	0 (0)	0.122
Sertraline	35 (1.2)	7 (1.9)	0.189	1 (0.5)	0.407	2 (1.7)	0.589
Tramadol	78 (2.7)	12 (3.2)	0.463	3 (1.6)	0.378	5 (4.3)	0.257
Venlafaxine	40 (1.4)	5 (1.3)	0.976	2 (1.1)	0.747	2 (1.7)	0.731
**CNS efficacy only**							
Fentanyl	99 (3.4)	6 (1.6)	**0.045**	2 (1.1)	0.078	3 (2.6)	0.634
Levetiracetam	58 (2.0)	3 (0.8)	0.083	7 (3.8)	0.063	3 (2.6)	0.628
Levodopa	82 (2.8)	17 (4.6)	**0.026**	9 (4.9)	0.071	0 (0)	0.063
Lorazepam	81 (2.8)	9 (2.4)	0.671	3 (1.6)	0.341	1 (0.9)	0.204
Pregabaline	161 (5.5)	25 (6.7)	0.260	6 (3.3)	0.177	6 (5.2)	0.883
Tilidine	110 (3.7)	15 (4.0)	0.753	3 (1.6)	0.122	3 (2.6)	0.503

Significant differences in unadjusted *p*-values are depicted by **bold** text.

CNS drugs most frequently taken were pregabaline (*n* = 161, 5.5%), (es) citalopram (*n* = 143, 4.9%), and mirtazapine (*n* = 139, 4.7%), whereas most frequent CYP2D6 substrates were metoprolol (*n* = 932, 31.7%), simvastatin (*n* = 610, 20.8%), and bisoprolol (*n* = 286, 9.7%).

Of the total cohort, 12.7% (*n* = 372) presented with drug-associated dizziness. Table 2 shows the characteristics of the study population presenting either with or without dizziness. Patients that presented to the ED with dizziness had more comorbidities and took more drugs suspected for causing ADR symptoms. The condition at discharge differed between the two groups without a linear trend.

**TABLE 2:** Characteristics of the study population stratified in presenting with or without dizziness.

	**Dizziness, *n* = 372**	**No dizziness, *n* = 2567**	***p*-value**
Age, median (IQR)	74 (59; 81)	73 (58; 80)	0.366
Sex (female), n (%)	180 (48.4)	1274 (49.6)	0.654
Comorbidity, median (IQR)^a^	5 (3; 8)	5 (3; 7)	**0.013**
Intake of drugs, median (IQR)	7 (4; 9)	7 (3; 10)	0.268
Suspected drugs, median (IQR)	2 (1; 3)	1 (1; 2)	**<0.001**
Intake any CYP2D6 substrate, n (%)	257 (69.1)	1638 (63.8)	**0.047**
CYP2D6 substrates, median (IQR)	1 (0; 2)	1 (0; 2)	**0.014**
CYP2D6 saturation, n (%)			0.098
No CYP2D6 saturation	115 (30.9)	929 (36.2)	
Moderate CYP2D6 saturation	218 (58.6)	1381 (53.8)	
Strong CYP2D6 saturation	39 (10.5)	257 (10.0)	
CYP2D6 saturation/inhibition, n (%)			0.170
No CYP2D6 saturation/inhibition	113 (30.4)	914 (35.6)	
Weak CYP2D6 saturation/inhibition	216 (58.1)	1350 (52.6)	
Moderate CYP2D6 saturation/inhibition	40 (10.8)	284 (11.1)	
Strong CYP2D6 saturation/inhibition	3 (0.8)	19 (0.7)	
Symptoms, median (IQR)	4 (3; 5)	2 (1; 3)	**<0.001**
*Other CNS ADR symptoms, n (%)*			
Syncope	53 (14.2)	130 (5.1)	**<0.001**
Headache	18 (4.8)	98 (3.8)	0.345
*Presenting along with dizziness, n (%)*			
Nausea	81 (21.8)	239 (9.3)	**<0.001**
General physical health deterioration	61 (16.4)	339 (13.2)	0.093
Syncope	53 (14.2)	130 (5.1)	**<0.001**
Dyspnea	52 (14.0)	352 (13.7)	0.889
Fall	45 (12.1)	126 (4.9)	**<0.001**
Seriousness, n (%)			0.494
Nonserious harm	48 (12.9)	269 (10.5)	
Hospitalization required	303 (81.5)	2123 (82.7)	
Life threatening	21 (5.6)	168 (6.5)	
Death	—	7 (0.3)	
Condition at discharge, n (%)			**0.010**
Recovered	15 (4.2)	128 (5.3)	
Not recovered	39 (10.8)	232 (9.6)	
Condition improved	303 (83.9)	1935 (80.0)	
Persistent harm	—	17 (0.7)	
Death	4 (1.1)	108 (4.5)	

Significant differences in unadjusted *p*-values are depicted by **bold** text.

a Information on comorbidities missing in *n* = 262 cases.

CNS: central nervous system; IQR: interquartile range.

No CYP2D6 saturation: number of CYP2D6 substrates = 0.

Moderate CYP2D6 saturation: number of CYP2D6 substrates = 1, 2.

Strong CYP2D6 saturation: number of CYP2D6 substrates ≥3.

No CYP2D6 saturation/inhibition: score = 0.

Weak CYP2D6 saturation/inhibition: score = 1, 2

Moderate CYP2D6 saturation/inhibition: score = 3, 4.

Strong CYP2D6 saturation/inhibition: score ≥5.

There was a significant difference in CYP2D6 substrate intake in patients with dizziness compared to patients without dizziness. Presenting with dizziness was positively associated with stronger CYP2D6 saturation but not with stronger CYP2D6 saturation/inhibition. Phenotyping results for polymorphic CYP enzymes are pictured in Supplementary Table S3.

Presenting with syncope was associated with dizziness whereas headache was not. Patients with dizziness had more additional ADR symptoms on ED admission. The five ADR symptoms seen most commonly together with dizziness were dyspnea (13.7%, *n* = 404), general physical health deterioration (13.6%, *n* = 400), nausea (10.9%, *n* = 320), syncope (6.2%, *n* = 183), and falls (5.8%, *n* = 171). Dizziness was significantly associated with nausea, syncope, and falls as concomitant ADR symptoms.

Patients that presented with or without dizziness took in median one CYP2D6 substrate (IQR 0; 2), even though differing significantly (*p* = 0.014). Using unadjusted regression analyses, the chance to present with drug-related dizziness to the ED increased with each CYP2D6 substrate taken by OR 1.11 [1.01–1.23] as shown in Table 3.

**TABLE 3:** Associations of CYP2D6 activity markers with drug-related dizziness.

	**Unadjusted OR [95% CI]**	**Age–sex adjusted OR [95% CI]** **^a^**	**Multi-adjusted OR [95% CI]** **^b^**
Number of CYP2D6 substrates taken	1.11 [1.01–1.23]	1.12 [1.01–1.24]	1.25 [1.10–1.43]
CYP2D6 saturation (yes)	1.27 [1.00–1.60]	1.29 [1.00–1.66]	1.54 [1.16–2.05]
CYP2D6 saturation/inhibition (yes)	1.27 [1.00–1.60]	1.28 [1.00–1.66]	1.54 [1.16–2.04]
Composed CYP2D6 activity (poor/intermediate)	1.59 [0.92–2.75]	1.56 [0.89–2.76]	1.77 [0.98–3.17]

a Model including age, sex, and number of CYP2D6 substrates taken or CYP2D6 saturation, CYP2D6 saturation/inhibition, or composed CYP2D6 activity (poor/intermediate).

b Model including age, sex, total number of drugs taken, and number of CYP2D6 substrates taken or CYP2D6 saturation, CYP2D6 saturation/inhibition, or composed CYP2D6 activity (poor/intermediate).

There was no statistically significant difference in the number of CYP2D6 substrate exposure in patients that presented with syncope or not (with syncope: median 1 (0; 2) vs. without syncope: 1 (0; 2), *p* = 0.067, and OR 1.11 [0.97–1.27]), although a tendency toward significance was observed. Patients that presented with headache took significantly less CYP2D6 substrates than those presenting without headache [0 (0; 1) vs. 1 (0; 2), *p* < 0.001, and OR 0.57 (0.45–0.71)].

These associations with CYP2D6 substrate exposure were likewise seen when excluding patients with dizziness (with syncope: 1 (0; 2) vs. without: 1 (0; 2), *p* = 0.143, OR 1.10 [0.94–1.30] and with headache: 0 (0; 1) vs. without: 1 (0; 2), *p* < 0.001, OR 0.54 [0.42–0.70], respectively).

**FIGURE 1:**
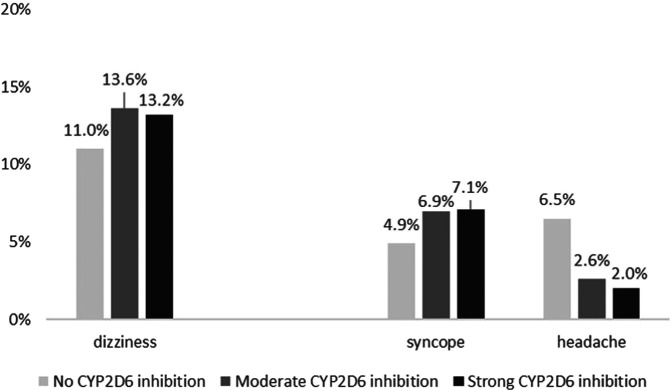
Association of CYP2D6 saturation by drug exposure on the occurrence of dizziness (*p* = 0.098), syncope (*p* = 0.043), and headache (*p* < 0.001).

In the total population, 35.5% of patients (*n* = 1044) took no CYP2D6 substrate (no CYP2D6 saturation), 54.4% (*n* = 1599) took one or two substrates (moderate CYP2D6 saturation), and 10.1% (*n* = 296) took three or more substrates of CYP2D6 (strong CYP2D6 saturation). Figure 1 shows the effect of CYP2D6 saturation by drug exposure on the occurrence of the frequent CNS ADR symptoms dizziness, syncope, and headache.

Of patients with strong CYP2D6 saturation, 13.2% (*n* = 39) presented with dizziness, with moderate CYP2D6 saturation 13.6% (*n* = 218), and without CYP2D6 saturation 11.0% (*n* = 114). CYP2D6 saturation score was not significantly associated with dizziness, but a tendency toward significance could be seen (*p* = 0.098) and the chance to present with drug-related dizziness was higher with CYP2D6 saturation by drug exposure (1.27 [1.00–1.60]). However, 7.1% (*n* = 21) of patients with strong CYP2D6 saturation presented with syncope, 6.9% (*n* = 111) with moderate CYP2D6 saturation, and 4.9% (*n* = 51) without CYP2D6 saturation with a linear association (*p* = 0.043, OR 1.46 [1.05–2.03]). Again, patients presenting with headache had commonly no CYP2D6 saturation by drug exposure (6.5%, *n* = 68) compared with moderate CYP2D6 saturation (2.6%, *n* = 42) and with strong CYP2D6 saturation (2.0%, *n* = 6) (*p* < 0.001, OR 0.37 [0.26–0.54]).

When excluding patients with dizziness, there remained only a tendency toward significance for a linear association with syncope [5.8% (*n* = 15) strong CYP2D6 saturation, 5.7% (*n* = 79) moderate CYP2D6 saturation, and 3.9% (*n* = 36) no CYP2D6 saturation, *p* = 0.062]. However, the same effect was seen for headache with stronger CYP2D6 saturation showing less headache [1.9% (*n* = 5) strong CYP2D6 saturation, 2.5% (*n* = 34) moderate CYP2D6 saturation, and 6.4% (*n* = 59) no CYP2D6 saturation, *p* < 0.001].

**FIGURE 2:**
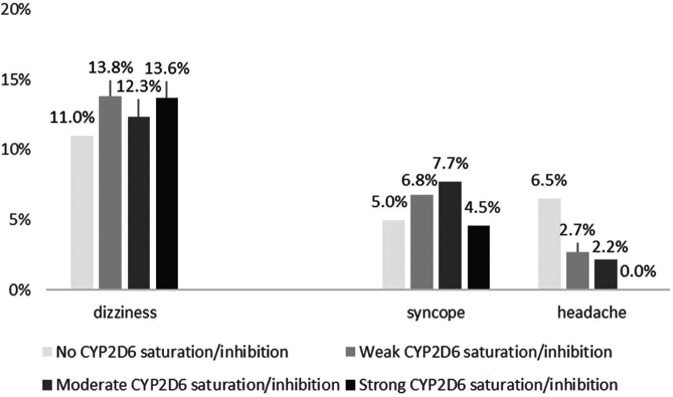
Association of CYP2D6 saturation/inhibition by drug exposure on the occurrence of dizziness (*p* = 0.170), syncope (*p* = 0.050), and headache (*p* < 0.001).

Combining CYP2D6 saturation and inhibition information, 34.9% of all patients (*n* = 1027) took neither a CYP2D6 substrate nor a CYP2D6 inhibitor (CYP2D6 saturation/inhibition score = 0), 53.3% (*n* = 1566) experienced weak, 11.0% (*n* = 324) moderate, and 0.7% (*n* = 22) strong saturation/inhibition as expressed by the CYP2D6 saturation/inhibition score. Figure 2 shows the effect of CYP2D6 saturation/inhibition by drug exposure on the occurrence of frequent CNS ADR symptoms.

Of patients with strong CYP2D6 saturation/inhibition, 0.8% (*n* = 3) presented with dizziness, with moderate 10.8% (*n* = 40), with weak 58.1% (*n* = 216), and without CYP2D6 saturation/inhibition 30.4% (*n* = 113) without a statistically significant linear trend (*p* = 0.170). However, the chance to present with drug-related dizziness was higher with CYP2D6 saturation/inhibition by drug exposure (1.27 [1.00–1.60]). Syncope was associated linearly with CYP2D6 saturation/inhibition (*p* = 0.050, and OR 1.42 [1.02–1.98]), while headache was again reversely associated with CYP2D6 saturation/inhibition (*p* < 0.001, and OR 0.38 [0.26–0.55]).

With exclusion of patients with dizziness, syncope was not any longer associated with CYP2D6 saturation/inhibition (*p* = 0.059), while the association for headache remained significant (*p* < 0.001).

**FIGURE 3:**
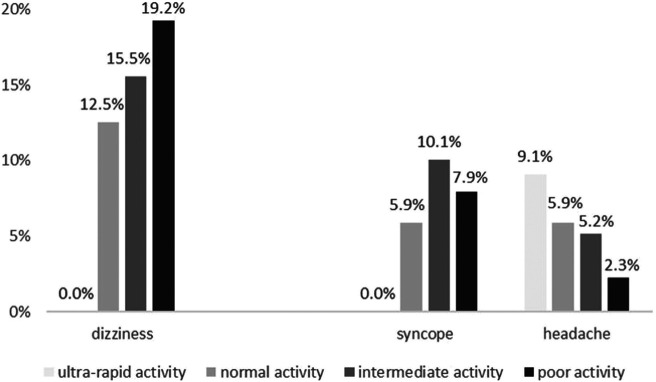
Association of CYP2D6 activity derived from combination of the genotype with CYP2D6 saturation by drug exposure on the occurrence of dizziness (*p* = 0.028), syncope (*p* = 0.483), and headache (*p* = 0.041).

Among the patients genotyped for CYP2D6 (*n* = 740), 6.6% (*n* = 49) were PM, 36.8% (*n* = 272) IM, 53.2% (*n* = 394) NM, and 3.4% (*n* = 25) UM as predicted by the genotype. Including additive effects of CYP2D6 saturation by drug exposure, 35.8% of patients (*n* = 265) were expected to show poor, 44.3% (*n* = 328) intermediate, 18.4% (*n* = 136) normal, and 1.5% (*n* = 11) ultrarapid activity of the CYP2D6 enzyme. No patient with composed predicted CYP2D6 ultrarapid activity presented with dizziness or syncope to the ED. Figure 3 shows the effect of composed CYP2D6 activity combining the genotype-predicted phenotype with CYP2D6 saturation by drug exposure on the occurrence of dizziness, syncope, and headache.

Of patients with poor composed CYP2D6 activity, 19.2% (*n* = 51) presented with dizziness, with intermediate CYP2D6 activity 15.5% (*n* = 51), and with normal CYP2D6 activity 12.5% (*n* = 17). Composed CYP2D6 activity was associated linearly with the occurrence of dizziness (*p* = 0.028) in the genotyped cohort. There was no linear association of composed CYP2D6 activity with syncope [7.9% (*n* = 21) with poor CYP2D6 activity, 10.1% (*n* = 33) with intermediate CYP2D6 activity, and 5.9% (*n* = 8) with normal CYP2D6 activity, *p* = 0.483]. On the other hand, there was a linear association with headache [2.3% (*n* = 6) poor CYP2D6 activity, 5.2% (*n* = 17) intermediate CYP2D6 activity, 5.9% (*n* = 8) normal CYP2D6 activity, and 9.1% (*n* = 1) ultrarapid activity, *p* = 0.041].

We found similar results for syncope when excluding patients with dizziness [syncope 5.1% (*n* = 11) poor CYP2D6 activity, 8.3% (*n* = 23) intermediate CYP2D6 activity, and 4.2% (*n* = 5) normal CYP2D6 activity, *p* = 0.761]. There was no significant linear association for headache with exclusion of patients with dizziness, even though a tendency toward significance could be seen [2.3% (*n* = 5) poor CYP2D6 activity, 5.4% (*n* = 15) intermediate CYP2D6 activity, 5.9% (*n* = 7) normal CYP2D6 activity, and 9.1% (*n* = 7) ultrarapid activity, *p* = 0.067].

Falls, a symptom frequently associated with dizziness, were associated with the number of CYP2D6 substrates taken and CYP2D6 saturation and CYP2D6 saturation/inhibition but not with composed CYP2D6 activity in the genotyped subsample. Nausea was not associated with any CYP2D6 activity marker (number of CYP2D6 substrates taken, CYP2D6 saturation and CYP2D6 saturation/inhibition scores, and composed CYP2D6 activity). Results are shown in the supplement (Supplementary Table S4, S5).

All four CYP2D6 activity markers were associated with experiencing drug-related dizziness causing ED admission, when adjusted for age, sex, and the total number of drugs taken (Model 2). Results of binary logistic regression analyses are shown in Table 3.

The effects of CYP2D6 activity markers on dizziness got higher when adjusting for the total number of drugs taken, age, and sex compared to only adjusting for age and sex. Effects increased from using the absolute number of CYP2D6 substrate exposure (OR 1.25 [1.10–1.43]) to the CYP2D6 saturation score (1.54 [1.16–2.05]), with comparable results for the CYP2D6 saturation/inhibition score (1.54 [1.16–2.04]) and to the composed CYP2D6 activity using additive effects of CYP2D6 substrate exposure and genotype (1.77 [0.98–3.17]) as CYP2D6 activity marker. However, while the OR increased, the confidence interval intersects with one showing that the genotyped cohort was slightly too small to reach statistical significance.

## Discussion

This study shows, to the best of our knowledge, for the first time an association of dizziness, an ADR symptom related to CNS function, with CYP2D6 activity leading to ED admission. This is a striking finding as it might point to a constitutive role of the polymorphic CYP2D6 enzyme on basic brain function.

Dizziness is a common clinical phenomenon that can be related to a wide variety of drugs (Sloane et al., 2001) and was the CNS ADR symptom most frequently seen in our dataset. Notably, roughly a third of patients took CNS drugs in our cohort, but more than 60% took CYP2D6 substrates. Thus, CYP2D6 substrates consisted of heterogeneous drug classes which did not always have only CNS main effects. Dizziness, as a rather unspecific symptom, can be caused or deteriorated by many drugs and is not exclusively linked to CYP2D6 substrates. However, effects of CYP2D6 activity markers on dizziness got higher when adjusting for the total number of drugs taken in logistic regression models. Another result that points out the role of CYP2D6 activity in vigilance-related ADR symptoms is the fact that other CNS side effects such as syncope did not correlate consistently with the CYP2D6 activity markers when excluding patients with dizziness. However, headache correlated reversely with CYP2D6 activity in the total population with higher activity associated with less drug-related headache. This could be explained by the higher amount of patients taking opioids and other drugs used to treat pain such as amitriptyline metabolized by CYP2D6. The group of patients with headache was quite small, and this effect should be analyzed more in depth with including symptoms correlating with headache. Therefore, further analyses would be needed.

Phenomenologically, dizziness is connected to alertness, attention, and vigilance. Our results are in line with the aforementioned fMRI studies showing an association of the CYP2D6 genotype on brain function connected with alertness and attention. However, in fMRI studies, conflicting results on the direction of sustained attention with lower or higher CYP2D6 activity were seen (Kirchheiner et al., 2011; Stingl et al., 2012; Viviani et al., 2020). Our study clearly points to lower CYP2D6 activity being associated with dizziness and thus reduced alertness and vigilance.

CYP2D6 might be an important enzyme for local serotonin and dopamine syntheses in the brain (Haduch and Daniel, 2018) and thereby influence cognitive function. Some drugs interacting with the serotonergic and dopaminergic system, potentially elevating serotonin and dopamine levels are known to frequently cause dizziness as an ADR such as antidepressants, antiemetics, and serotonergic opioids. Noteworthy, although no study center here had a psychiatric ward, a third of all patients in our study took a drug with intended CNS efficacy. However, many other drugs that are not designed to have a CNS main effect are known to pass the blood–brain barrier such as the beta-blocker metoprolol or the antiarrhythmic amiodarone. Therefore, they might potentially inhibit brain CYP2D6 and provoke ADR symptoms directly in the CNS. CYP2D6 is known to be prone for phenocopying by drug–drug interactions as well as autophenocopying by reducing its own metabolism (Gardiner and Begg, 2006). Concordantly, metabolism by CYP2D6 was described to be easily inhibited (Sindrup et al., 1992; Taylor et al., 2006). This might point to a saturation of brain CYP2D6 with higher CYP2D6 substrate exposure, potentially affecting local serotonin and dopamine syntheses and could explain the occurrence of drug-associated dizziness with stronger CYP2D6 saturation by substrate exposure and genotype. Notably, effects of pure CYP2D6 saturation and combined effects of saturation and inhibition were comparable in our analysis.

Dizziness occurred frequently with other ADR symptoms in our dataset. We documented all ADR-associated symptoms on ED arrival, and therefore, dizziness might not always have been the primary symptom leading to ED presentation. However, even if not the primary symptom, but part of a symptom complex, we could show an association that points to a higher vulnerability for drug-associated dizziness in case of lower CYP2D6 activity. Furthermore, we analyzed concomitant ADR symptoms. While only dizziness and not those concomitant symptoms were consistently associated with CYP2D6 activity markers, those sensitivity analyses might underline the importance of CYP2D6 activity on drug-related dizziness. However, some concomitant ADR symptoms are likely to occur in the context of dizziness and reduced vigilance. As an example, drug-related falls were associated with CYP2D6 substrate exposure and with CYP2D6 saturation. This could be seen in line with many drugs metabolized by CYP2D6 that are considered fall-risk increasing (Just et al., 2017). This finding could not be seen in the genotyped subsample. While drug-associated falls are a multifactorial phenomenon, the genotype might not have a substantial impact on its occurrence. However, the sample size was rather small to show any effects with only eleven patients with poor calculated CYP2D6 activity presenting with a drug-associated fall.

The results of this study are at least partially generalizable. CYP2D6 is expected to metabolize up to 25% of commonly taken drugs (Ingelman-Sundberg et al., 2007). Thus, CYP2D6 saturation is a frequent phenomenon and may predict CNS CYP2D6 activity even more than the genotype. In our cohort with a median intake of seven drugs, the vast majority of patients took at least one CYP2D6 substrate. As our population was enrolled in EDs, it covers an intersectoral interface of ambulatory and hospital care patients. Therefore, our study population represents an important population of mainly multi-medicated, older adults having a relevant impact on health-care utilization.

Strength of this study is first of all the large sample size of prospectively enrolled, well-characterized ADR cases, including genotypes for a large subsample. Secondly, to the best of our knowledge, this is the first study to analyze the impact of CYP2D6 activity on ADRs considering CYP2D6 saturation by concomitant substrate exposure and, in a smaller subsample, the additive effects of substrate exposure and genotype. While the use of pharmacogenetics is expected to increase drug safety by optimizing the individual drug treatment (Evans and Relling, 2004), data on the impact of pharmacogenetics on the occurrence of ADRs are sparse and the clinical importance of genotype–phenotype discordance is unclear (Klomp et al., 2020). Therefore, our study adds an important piece to this puzzle.

Our study has potential limitations. We did not differentiate between CYP2D6 major or minor substrates for calculation of CYP2D6 saturation. Likewise, we assumed additive effects of saturation and inhibition. Thus, we built these analyses on assumptions testing linearity of CYP2D6 saturation, saturation/inhibition, and composed activity. In addition, we did not characterize the possibility to pass the blood–brain barrier. On the other hand, these classifications are usually derived from *in vitro* studies and the effect *in vivo*, in particular in multi-medicated, older adults, might differ. We were not able to check patients’ medication adherence, for example, by measuring concentrations of drugs in blood or plasma of patients. However, our study is in line with a signal detection approach in pharmacovigilance, where the suspected case of an ADR is already a signal documented in surveillance databases such as EudraVigilance or the WHO database.

Notably, the genotyped subsample might have diverging limitations. For genotyping, we needed testing and written informed consent which was not possible to obtain from all patients for logistic and ethical reasons. Therefore, ADRs resulting in death and loss of consciousness are missing in this subsample. However, as genotyping was probably easier in hospitalized patients with extended hospitalization time, this subsample represents a group of serious ADRs. The extrapolation of the phenotype from the genotype represents current knowledge on functional variants in a constantly developing field. Limitations of CYP2D6 phenotype extrapolation based on genotypes are discussed more in detail elsewhere (Just et al., 2020b). In addition, genetic NM may not represent a homogenous population and some might convert to IM and some to PM by the same drug exposure (Storelli et al., 2018). Therefore, phenotyping would be needed for a distinct analysis. However, the frequencies of genetic PM and corresponding calculated CYP2D6 activity in our study are comparable with other data on CYP2D6 phenoconversion analyzing genotype–phenotype correlations (Preskorn et al., 2013; Lisbeth et al., 2016). In addition, other drug–drug interactions and drug–gene interactions might be of relevance that was not tested here. Thus, the impact of other polymorphic CYP enzymes would need further investigation.

In conclusion, for the first time, this study shows an impact of CYP2D6 activity on the occurrence of the clinically relevant CNS ADR symptom dizziness. As this is shown for the first time, it would need replication in a different dataset. While evidence points to an important role of CYP2D6 activity on brain function, alertness, and serotonergic and dopaminergic pathways, this study may underline the clinical relevance of CYP2D6 activity affected by genotype and drug exposure on drug-associated dizziness. As dizziness is a frequent symptom in ADR-related ED admissions, this study is of importance to understand individualized ADR risks in older, multi-medicated patients.

## Data Availability

The data analyzed in this study are subject to the following licenses/restrictions: The datasets analyzed during the current study are available from the corresponding author on reasonable request. Requests to access these datasets should be directed to jstingl@ukaachen.de.
