# Supplementary material for: CYP2D6 in the Brain: Potential Impact on Adverse Drug Reactions in the Central Nervous System—Results From the ADRED Study
Source: Front Pharmacol. 2021 May 7;12:624104. doi: 10.3389/fphar.2021.624104 (PMC8138470; doi:10.3389/fphar.2021.624104)
Supplement: Supplementary file 1 [file Table1.DOCX]

**Supplement 1**: Classification of drugs frequently suspected (in >0.3% of cases) or taken (in >3% of cases) by the population.

| **Substance** | **CNS efficacy intended** | **Major CYP2D6 substrate** | **Minor CYP2D6 substrate** | **Any CYP2D6 substrate** |
| --- | --- | --- | --- | --- |
| Acetylsalicylic acid | - | - | - | - |
| Allopurinol | - | - | - | - |
| Amiodarone | - | - | x | x |
| Amitriptyline | x | x |  | x |
| Amlodipine | - | - | - | - |
| Apixaban | - | - | - | - |
| Atorvastatin | - | - | - | - |
| Bisoprolol | - | - | x | x |
| Candesartan | - | - | - | - |
| Carboplatin | - | - | - | - |
| Carvedilol | - | x | - | x |
| Citalopram | x | - | x | x |
| Clopidogrel | - | - | - | - |
| Colecalciferol | - | - | - | - |
| Diclofenac | - | - | - | - |
| Digitoxine | - | - | - | - |
| Duloxetine | x | x | - | x |
| Enoxaparin | - | - | - | - |
| Escitalopram | x | - | x | x |
| Fentanyl | x | - | - | - |
| Fluorouracil | - | - | - | - |
| Folic acid | - | - | - | - |
| Furosemide | - | - | - | - |
| Hydrochlorothiazide | - | - | - | - |
| Ibuprofen | - | - | - | - |
| Insulin (human) | - | - | - | - |
| Insulin glargin | - | - | - | - |
| Levetiracetam | x | - | - | - |
| Levodopa | x | - | - | - |
| Levothyroxine | - | - | - | - |
| Lorazepam | x | - | - | - |
| Macrogol | - | - | - | - |
| Metamizole/ Dipyrone | - | - | - | - |
| Metformin | - | - | - | - |
| Metoclopramide | - | x | - | x |
| Metoprolol | - | x | - | x |
| Mirtazapine | x | - | x | x |
| Omeprazole | - | - | - | - |
| Oxycodone | x | - | x | x |
| Pantoprazole | - | - | - | - |
| Phenprocoumon | - | - | - | - |
| Prednisolone | - | - | - | - |
| Pregabaline | x | - | - | - |
| Quetiapine | x | - | x | x |
| Ramipril | - | - | - | - |
| Risperidone | x | x | - | x |
| Rivaroxaban | - | - | - | - |
| Salbutamol | - | - | - | - |
| Sertraline | x | - | x | x |
| Simvastatin | - | - | x | x |
| Sitagliptin | - | - | - | - |
| Spironolactone | - | - | - | - |
| Tamsulosin | - | x | - | x |
| Tilidine | x | - | - | - |
| Tiotropiumbromide | - | - | x | x |
| Torasemide/Torsemide | - | - | - | - |
| Tramadol | x | x | - | x |
| Valsartan | - | - | - | - |
| Venlafaxine | x | x | - | x |
| Xipamide | - | - | - | - |
