# Supplementary material for: CYP2D6 in the Brain: Potential Impact on Adverse Drug Reactions in the Central Nervous System—Results From the ADRED Study
Source: Front Pharmacol. 2021 May 7;12:624104. doi: 10.3389/fphar.2021.624104 (PMC8138470; doi:10.3389/fphar.2021.624104)
Supplement: Supplementary file 2 [file Table2.DOCX]

**Supplement 2**: CYP2D6 activity composed of the genotype-predicted phenotype and CYP2D6 saturation by substrate exposure.

| **Genotype-predicted CYP2D6 phenotype** | **No CYP2D6 saturation** | **Moderate CYP2D6 saturation** | **Strong CYP2D6 saturation** |
| --- | --- | --- | --- |
| **Poor metabolizer** | poor activity | poor activity | poor activity |
| **Intermediate metabolizer** | intermediate activity | poor activity | poor activity |
| **Normal metabolizer** | normal activity | intermediate activity | poor activity |
| **Ultra-rapid metabolizer** | ultra-rapid activity | normal activity | intermediate activity |

No CYP2D6 saturation: no CYP2D6 substrate was taken

Moderate CYP2D6 saturation: one or two CYP2D6 substrates were taken

Strong CYP2D6 saturation: three or more CYP2D6 substrates were taken.
