# Supplementary material for: CYP2D6 in the Brain: Potential Impact on Adverse Drug Reactions in the Central Nervous System—Results From the ADRED Study
Source: Front Pharmacol. 2021 May 7;12:624104. doi: 10.3389/fphar.2021.624104 (PMC8138470; doi:10.3389/fphar.2021.624104)
Supplement: Supplementary file 3 [file Table5.DOCX]

**Supplement 5:** Association of CYP2D6 activity markers with nausea (frequently associated with dizziness).

|  | **Total population: N=2939** | | | **Without dizziness: n=2567** | | | |
| --- | --- | --- | --- | --- | --- | --- | --- |
|  | **Nausea, n=320** | **No nausea, n=2619** | **p-value** | **Nausea, n=239** | | **No nausea, n=2328** | **p-value** |
| CYP2D6 substrates, median (IQR) | 1 (0; 2) | 1 (0; 2) | 0.566 | 1 (0; 2) | | 1 (0; 2) | 0.094 |
| CYP2D6 saturation, n (%) |  |  | 0.599 |  | |  | 0.146 |
| No CYP2D6 saturation | 114 (35.6) | 930 (35.5) |  | 95 (39.7) | | 834 (35.8) |  |
| Moderate CYP2D6 saturation | 179 (55.9) | 1420 (54.2) |  | 125 (52.3) | | 1256 (54.0) |  |
| Strong CYP2D6 saturation | 27 (8.4) | 269 (10.3) |  | 19 (7.9) | | 238 (10.2) |  |
| CYP2D6 saturation/inhibition, n (%) |  |  | 0.461 |  | |  | 0.105 |
| No CYP2D6 saturation/inhibition | 112 (35.0) | 915 (34.9) |  | 94 (39.3) | | 820 (35.2) |  |
| Weak CYP2D6 saturation/inhibition | 177 (55.3) | 1389 (53.0) |  | 123 (51.5) | | 1227 (52.7) |  |
| Moderate CYP2D6 saturation/inhibition | 30 (9.4) | 294 (11.2) |  | 21 (8.8) | | 263 (11.3) |  |
| Strong CYP2D6 saturation/inhibition | 1 (0.3) | 21 (0.8) |  | 1 (0.4) | | 18 (0.8) |  |
|  | **Genotyped subgroup: n=740** | | | **Without dizziness: n=621** | | | |
|  | **Nausea, n=103** | **No nausea, n=637** |  | **Nausea, n=74** | **No nausea, n=547** | |  |
| Composed CYP2D6 activity, n (%) |  |  | 0.395 |  |  | | 0.712 |
| Ultra-rapid activity | 2 (1.9) | 9 (1.4) |  | 2 (2.7) | 9 (1.6) | |  |
| Normal activity | 18 (17.5) | 118 (18.5) |  | 14 (18.9) | 105 (19.2) | |  |
| Intermediate activity | 40 (38.8) | 288 (45.2) |  | 29 (39.2) | 248 (45.3) | |  |
| Poor activity | 43 (41.7) | 222 (34.9) |  | 29 (39.2) | 185 (33.8) | |  |
