# Supplementary material for: CYP2D6 in the Brain: Potential Impact on Adverse Drug Reactions in the Central Nervous System—Results From the ADRED Study
Source: Front Pharmacol. 2021 May 7;12:624104. doi: 10.3389/fphar.2021.624104 (PMC8138470; doi:10.3389/fphar.2021.624104)
Supplement: Supplementary file 4 [file Table3.DOCX]

**Supplement 3**: Genotype-predicted phenotypes of polymorphic CYP-enzymes in the genotyped cohort (N=776) stratified in presenting with or without dizziness with unadjusted p-values.

|  | **Dizziness, n=121** | **No dizziness, n=637** | **p-value** |
| --- | --- | --- | --- |
| CYP2D6 phenotype, n (%) |  |  | 0.396 |
| Poor metabolizer | 8 (6.7) | 41 (6.6) |  |
| Intermediate metabolizer | 43 (36.1) | 229 (36.9) |  |
| Normal metabolizer | 67 (56.3) | 327 (52.7) |  |
| Ultra-rapid metabolizer | 1 (0.8) | 24 (3.9) |  |
| CYP2C9 phenotype, n (%) |  |  | 0.601 |
| Poor metabolizer | 4 (3.3) | 23 (3.6) |  |
| Intermediate metabolizer | 43 (35.2) | 196 (30.6) |  |
| Normal metabolizer | 75 (61.5) | 421 (65.8) |  |
| CYP2C19 phenotype, n (%) |  |  | 0.153 |
| Poor metabolizer | 2 (1.7) | 26 (4.1) |  |
| Intermediate metabolizer | 24 (19.8) | 177 (27.8) |  |
| Normal metabolizer | 59 (48.8) | 248 (38.9) |  |
| Rapid metabolizer | 32 (26.4) | 160 (25.1) |  |
| Ultra-rapid metabolizer | 4 (3.3) | 26 (4.1) |  |

For CYP2D6 n=36, for CYP2C9 n=14, and for CYP2C19 n=18 missing cases.
