# Supplementary material for: CYP2D6 in the Brain: Potential Impact on Adverse Drug Reactions in the Central Nervous System—Results From the ADRED Study
Source: Front Pharmacol. 2021 May 7;12:624104. doi: 10.3389/fphar.2021.624104 (PMC8138470; doi:10.3389/fphar.2021.624104)
Supplement: Supplementary file 5 [file Table4.DOCX]

**Supplement 4:** Association of CYP2D6 activity markers with falls (frequently associated with dizziness).

|  | **Total population: N=2939** | | | **Without dizziness: n=2567** | | |
| --- | --- | --- | --- | --- | --- | --- |
|  | **Fall, n=171** | **No fall, n=2768** | **p-value** | **Fall, n=126** | **No fall, n=2441** | **p-value** |
| CYP2D6 substrates, median (IQR) | 1 (0; 2) | 1 (0; 2) | **0.013** | 1 (0; 2) | 1 (0; 2) | **0.040** |
| CYP2D6 saturation, n (%) |  |  | **0.020** |  |  | **0.042** |
| No CYP2D6 saturation | 50 (29.2) | 994 (35.9) |  | 38 (30.2) | 891 (36.5) |  |
| Moderate CYP2D6 saturation | 96 (56.1) | 1503 (54.3) |  | 69 (54.8) | 1312 (53.7) |  |
| Strong CYP2D6 saturation | 25 (14.6) | 271 (9.8) |  | 19 (15.1) | 238 (9.8) |  |
| CYP2D6 saturation/inhibition, n (%) |  |  | **0.029** |  |  | **0.039** |
| No CYP2D6 saturation/inhibition | 49 (28.7) | 978 (35.3) |  | 37 (29.4) | 877 (35.9) |  |
| Weak CYP2D6 saturation/inhibition | 95 (55.6) | 1471 (53.1) |  | 68 (54.0) | 1282 (52.5) |  |
| Moderate CYP2D6 saturation/inhibition | 25 (14.6) | 299 (10.8) |  | 19 (15.1) | 265 (10.9) |  |
| Strong CYP2D6 saturation/inhibition | 2 (1.2) | 20 (0.7) |  | 2 (1.6) | 17 (0.7) |  |
|  | **Genotyped subgroup: n=740** | | | **Without dizziness: n=621** | | |
|  | **Fall, n=36** | **No fall, n=704** |  | **Fall, n=22** | **No fall, n=599** |  |
| Composed CYP2D6 activity, n (%) |  |  | 0.963 |  |  | 0.691 |
| Ultra-rapid activity | 0 | 11 (1.6) |  | 0 (0) | 11 (1.8) |  |
| Normal activity | 6 (16.7) | 130 (18.5) |  | 4 (18.2) | 115 (19.2) |  |
| Intermediate activity- | 19 (52.8) | 309 (43.9) |  | 10 (45.5) | 267 (44.6) |  |
| Poor activity | 11 (30.6) | 254 (36.1) |  | 8 (36.4) | 206 (34.4) |  |
